# The White Matter Rounds experience: The importance of a multidisciplinary network to accelerate the diagnostic process for adult patients with rare white matter disorders

**DOI:** 10.3389/fneur.2022.928493

**Published:** 2022-07-25

**Authors:** Yu Tong Huang, Paul S. Giacomini, Rami Massie, Sunita Venkateswaran, Anne-Marie Trudelle, Giulia Fadda, Maryam Sharifian-Dorche, Hayet Boudjani, Laurence Poliquin-Lasnier, Laura Airas, Alexander W. Saveriano, Matthias Georg Ziller, Elka Miller, Claudia Martinez-Rios, Nagwa Wilson, Jorge Davila, Carolina Rush, Erin E. Longbrake, Giulia Longoni, Gabrielle Macaron, Geneviève Bernard, Donatella Tampieri, Jack Antel, Bernard Brais, Roberta La Piana

**Affiliations:** ^1^Department of Neurology and Neurosurgery, Montreal Neurological Institute, McGill University, Montreal, QC, Canada; ^2^Department of Pediatrics, Division of Neurology, CHEO, University of Ottawa, Ottawa, ON, Canada; ^3^Department of Neurology, CHU of Québec-Université Laval, Quebec, QC, Canada; ^4^Department of Neurology, Maisonneuve-Rosemont Hospital, Université de Montréal, Montreal, QC, Canada; ^5^Clinique Neuro-Outaouais, Gatineau, QC, Canada; ^6^Division of Clinical Neurosciences, Turku University Hospital and University of Turku, Turku, Finland; ^7^Department of Neurology, St. Mary's Hospital, Montreal, QC, Canada; ^8^Department of Medical Imaging, CHEO, University of Ottawa, Ottawa, ON, Canada; ^9^Division of Neurology, Neuroscience Department, University of Ottawa, Ottawa, ON, Canada; ^10^Department of Neurology, Yale MS Center, Yale School of Medicine, North Haven, CT, United States; ^11^Department of Pediatrics, Division of Neurology, The Hospital for Sick Children, University of Toronto, Toronto, ON, Canada; ^12^Department of Neurology, Hotel Dieu de France Hospital, Saint Joseph University, Beirut, Lebanon; ^13^Department of Specialized Medicine, Division of Medical Genetics, McGill University Health Center, Montreal, QC, Canada; ^14^Child Health and Human Development Program, Research Institute of the McGill University Health Center, Montreal, QC, Canada; ^15^Departments of Pediatrics and Human Genetics, McGill University, Montreal, QC, Canada; ^16^Department of Diagnostic Radiology, Kingston Health Science Centre, Queen's University, Kingston, ON, Canada; ^17^Department of Diagnostic Radiology, McGill University, Montreal, QC, Canada

**Keywords:** leukodystrophies, white matter diseases, multiple sclerosis, multidisciplinary (care or team), online meeting, rare diseases

## Abstract

**Introduction:**

Adult genetic leukoencephalopathies are rare neurological disorders that present unique diagnostic challenges due to their clinical and radiological overlap with more common white matter diseases, notably multiple sclerosis (MS). In this context, a strong collaborative multidisciplinary network is beneficial for shortening the diagnostic odyssey of these patients and preventing misdiagnosis. The White Matter Rounds (WM Rounds) are multidisciplinary international online meetings attended by more than 30 physicians and scientists from 15 participating sites that gather every month to discuss patients with atypical white matter disorders. We aim to present the experience of the WM Rounds Network and demonstrate the value of collaborative multidisciplinary international case discussion meetings in differentiating and preventing misdiagnoses between genetic white matter diseases and atypical MS.

**Methods:**

We retrospectively reviewed the demographic, clinical and radiological data of all the subjects presented at the WM Rounds since their creation in 2013.

**Results:**

Seventy-four patients (mean age 44.3) have been referred and discussed at the WM Rounds since 2013. Twenty-five (33.8%) of these patients were referred by an MS specialist for having an atypical presentation of MS, while in most of the remaining cases, the referring physician was a geneticist (23; 31.1%). Based on the WM Rounds recommendations, a definite diagnosis was made in 36/69 (52.2%) patients for which information was available for retrospective review. Of these diagnosed patients, 20 (55.6%) had a genetic disease, 8 (22.2%) had MS, 3 (8.3%) had both MS and a genetic disorder and 5 (13.9%) had other non-genetic conditions. Interestingly, among the patients initially referred by an MS specialist, 7/25 were definitively diagnosed with MS, 5/25 had a genetic condition (e.g., X-linked adrenoleukodystrophy and hereditary small vessel diseases like Cerebral Autosomal Dominant Arteriopathy with Subcortical Infarcts and Leukoencephalopathy (CADASIL) and *COL4A1*-related disorder), and one had both MS and a genetic demyelinating neuropathy. Thanks to the WM Rounds collaborative efforts, the subjects who currently remain without a definite diagnosis, despite extensive investigations performed in the clinical setting, have been recruited in research studies aimed at identifying novel forms of genetic MS mimickers.

**Conclusions:**

The experience of the WM Rounds Network demonstrates the benefit of collective discussions on complex cases to increase the diagnostic rate and decrease misdiagnosis in patients with rare or atypical white matter diseases. Networks of this nature allow physicians and scientists to compare and share information on challenging cases from across the world, provide a basis for future multicenter research studies, and serve as model for other rare diseases.

## Introduction

Up to 25% of patients with rare diseases experience a diagnostic odyssey lasting between 5 and 30 years. During this time, they undergo numerous specialist consultations along with several imaging, laboratory and genetic investigations. Even then, misdiagnoses have been reported in 40% of patients and many of them remain without a definite diagnosis (1).

Genetic leukoencephalopathies are a heterogenous group of rare diseases of the CNS white matter. While they were previously mainly reported in children, cases of adult-onset genetic leukoencephalopathies have been increasingly described in the past decade (2–4). Each single form is rare (highest prevalence <1:20,000) (5–7), but collectively their incidence may be close to that of multiple sclerosis (MS), the most common demyelinating disease in adults (8, 9). Adult genetic leukoencephalopathies have a wide spectrum of clinical and radiological presentations, that frequently overlap with acquired inflammatory, infectious and vascular white matter disorders (10, 11). The differential diagnosis with MS, particularly primary progressive (PPMS) and atypical forms, is especially challenging given that several genetic leukoencephalopathies can present as MS mimickers (12–14). To further complicate the diagnostic process, as with other rare diseases, many genes causing adult genetic leukoencephalopathies are still unknown (10). Specifically, 40–70% of adult patients are likely to remain without a definite diagnosis despite extensive testing (2–4). As patients spend years without an answer, they do not get disease-specific care, may receive inappropriate treatment, cannot receive genetic counseling, and may experience psychological distress. In this context, multidisciplinary networks of specialists are beneficial for shortening the diagnostic odyssey, sharing available knowledge, and identifying optimal care for each patient, all while requiring minimal resources.

The White Matter Rounds (WM Rounds) is a multidisciplinary network of physicians and scientists from North America, Europe, and the Middle East, who have gathered monthly since November 2013 to discuss challenging or atypical clinical cases with white matter disorders. Participants are mainly experts on genetic white matter disorders or MS. This allows for collective discussions on complex patient cases, sharing of experience and knowledge, improvement of patient workups and diagnoses, and recruitment of patients for research.

Here, we present the WM Rounds' experience and its usefulness to increase the diagnostic yield of patients with rare white matter disorders, reduce the risk of misdiagnosis, and provide the basis for joint research studies.

## Context and methods

**Figure 1:**
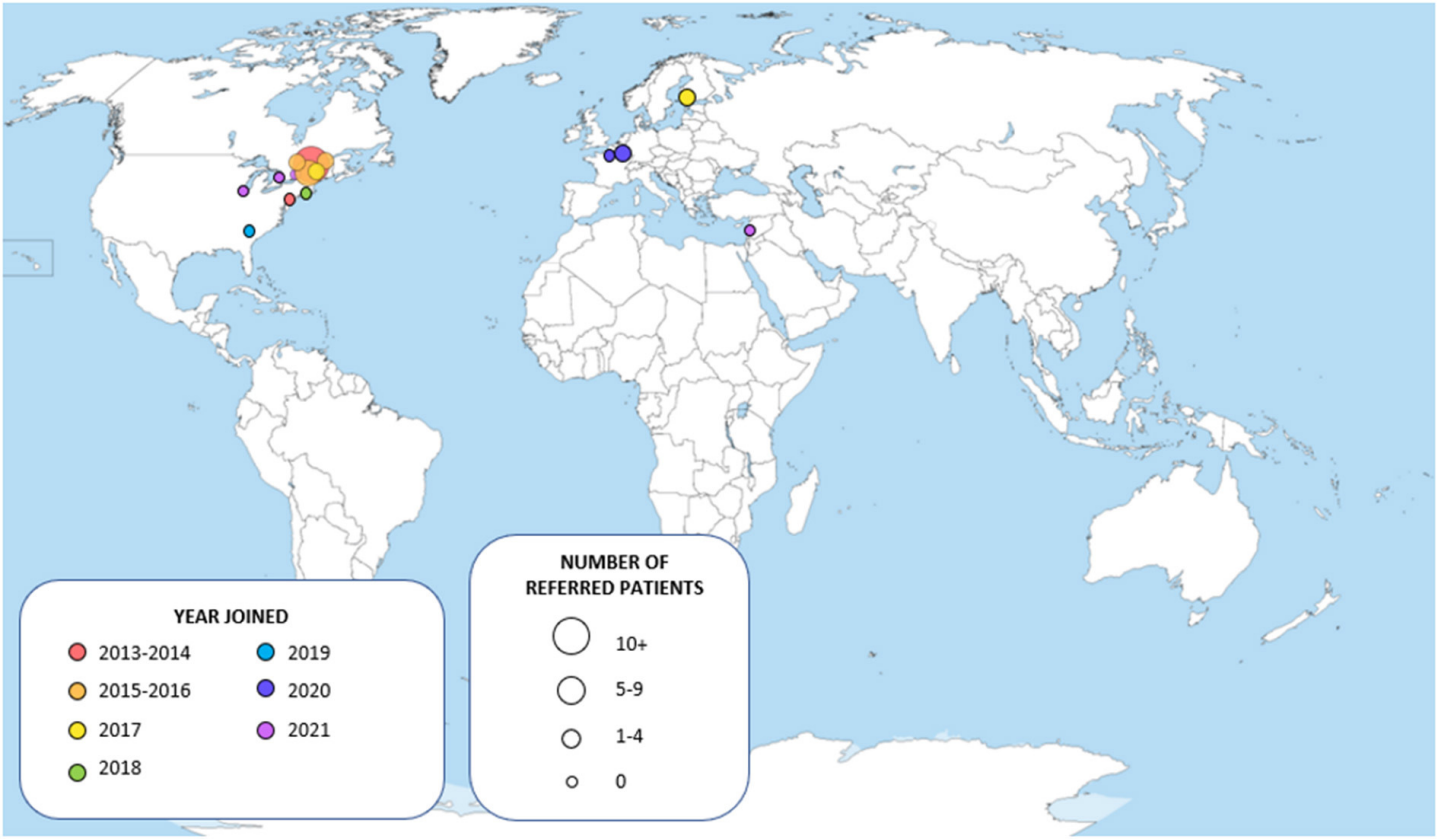
Map of the centers participating to the White Matter Rounds.

Since their creation in November 2013 at the Montreal Neurological Institute and Hospital (McGill University), the WM Rounds have grown into a network of 15 participating centers (Figure 1) with 30–35 regular attendees among which neurologists, geneticists, neuroradiologists and trainees. The meetings are held virtually to allow remote participation from all centers. To ensure confidentiality, patient information is anonymized, and shared data is further protected through the secured McGill University network. On three occasions, patients have attended the rounds after consent was obtained by the referring physician.

At each WM Rounds a maximum of two patients are presented to ensure enough time for discussion and team recommendations. Attendees can present patients for one of the following reasons: unknown diagnosis OR atypical clinical and/or radiological presentation of a known genetic or acquired white matter disorder. The referring physician presents the family and clinical histories, pertinent findings on examination of the patient, as well as the results of the diagnostic investigations, and other relevant information. Given the central role of white matter abnormalities interpretation, the available brain imaging studies are reviewed collegially and discussed. Attending members are invited to present follow-ups on their cases to inform the network about the diagnosis, or the lack thereof, in which case, it can be re-discussed, and other recommendations can be made.

We collected the anonymized subjects' age, sex, family history, clinical presentation, and laboratory test results presented by the referring physician, as well as the subspecialty of the presenting physician and center. In addition, we enter in our database the WM Rounds interpretation of the reviewed neuroimaging exams and the final recommendations following the discussion. For this project, we further contacted the referring physicians to obtain any information not available at the time of the WM Rounds presentation and to follow up on the patients' diagnostic outcomes.

## Results

### Cohort characteristics and demographics

**Figure 2:**
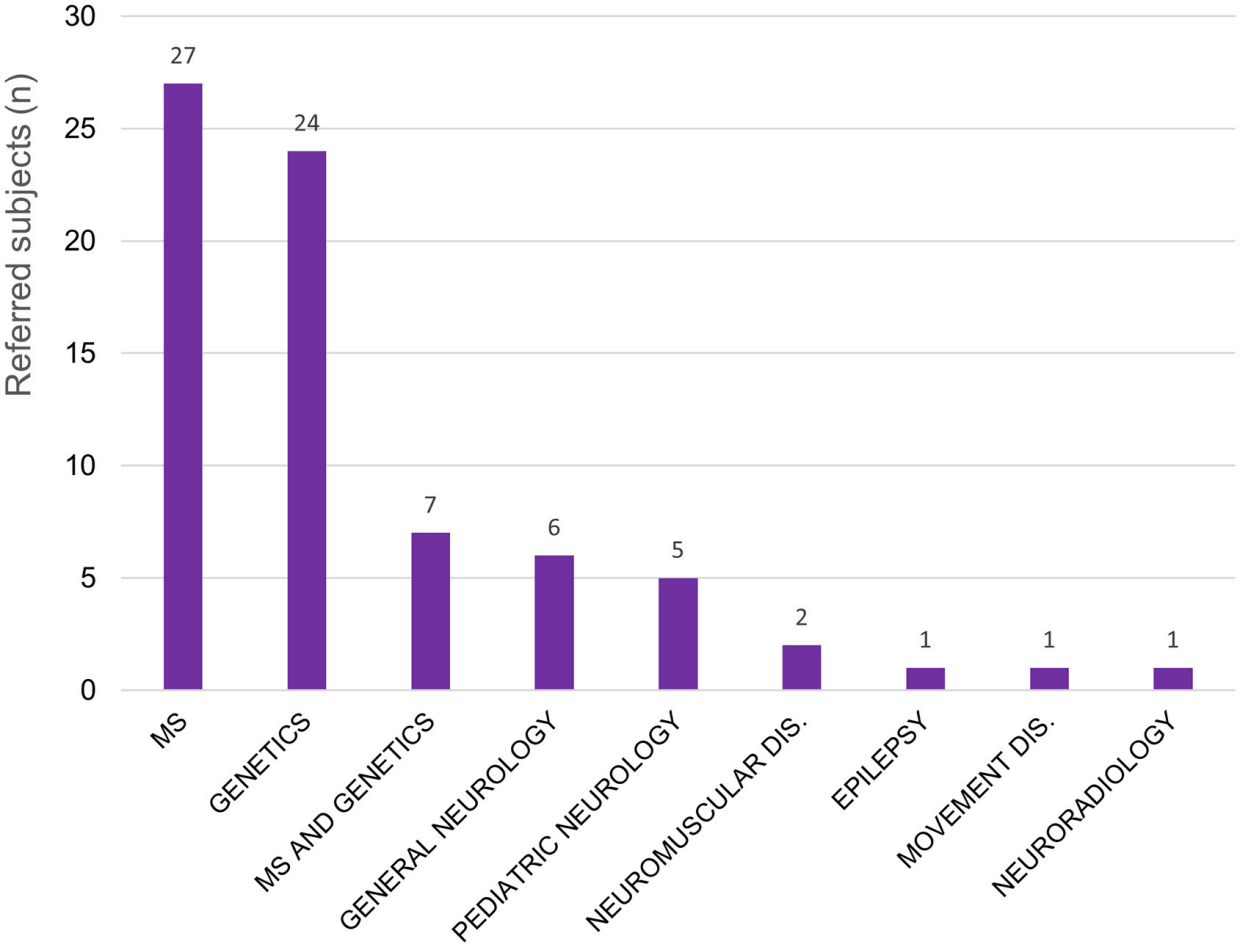
Patients discussed at the WM Rounds (total 74) according to the subspecialty of the referral physician. MS, multiple sclerosis; Dis, disorders.

A total of 74 patients have been presented and discussed at the WM Rounds since their launch in 2013 (Table 1); 13 subjects (17.6%; 13/74) were presented at more than one meeting for change in their clinical presentation or to re-discuss the diagnostic plan. Figure 1 illustrates the proportion of patients referred by each participating site; most patients were from centers in the province of Quebec, Canada, where the host site is located, and Eastern Ontario. For most of the patients, the referring physician was an MS specialist (25/74, 33.8%), a neurologist with expertise in genetics (23/74, 31.1%), or both an MS specialist and a neurologist with expertise in genetics collaborating on the same case (7/74; 9.5%) (Figure 2).

**Table 1:** Demographic and clinical features of the 74 subjects presented at the WM Rounds.

**Characteristics**	***n***	**%**
Total patients	74	
Female	37	50.0
Male	30	40.5
Sex unknown	7	9.5
Age (years)
Mean	44.3	
Median	45.0	
Range	12–75	
Duration of disease before diagnosis
Mean	11	
Range	0–41	
Family History
Consanguinity	2	2.7
Other affected family members	25	33.8
Clinical Presentation
Cerebellar signs	34	45.9
Cognitive/Psychiatric	27	36.5
Headache/Migraine	22	29.7
Visual	22	29.7
Sensory	12	16.2
Pyramidal signs	11	14.9
Neurogenic (bladder and bowel)	8	10.8
Numbness	8	10.8
Auditory	9	12.2
Neuropathy	6	8.1
Spastic paraparesis	5	6.8
Seizure	4	5.4
Developmental delay	3	4.1

The majority of patients [52/74 (70.3%)] were referred to the WM Rounds because of unknown diagnosis, with the goal of receiving inputs from the network about diagnostic hypotheses and plan. Twenty-two patients (29.7%) were referred while having already a working diagnosis, but for which the clinical presentation (nine subjects) or imaging findings (13 subjects) were atypical or novel; the discussions were then centered around the likelihood of the presumptive diagnosis, and thus expanding its phenotype and differential diagnosis.

### Clinical and radiological features

Table 1 reports the predominant clinical features of the presented patients. The most frequent clinical presentations were cerebellar signs (34/74; 45.9%), cognitive decline and/or psychiatric symptoms (27/74; 36.5%), headaches/migraines (22/74; 29.7%) and visual symptoms (22/74; 29.7%).

**Figure 3:**
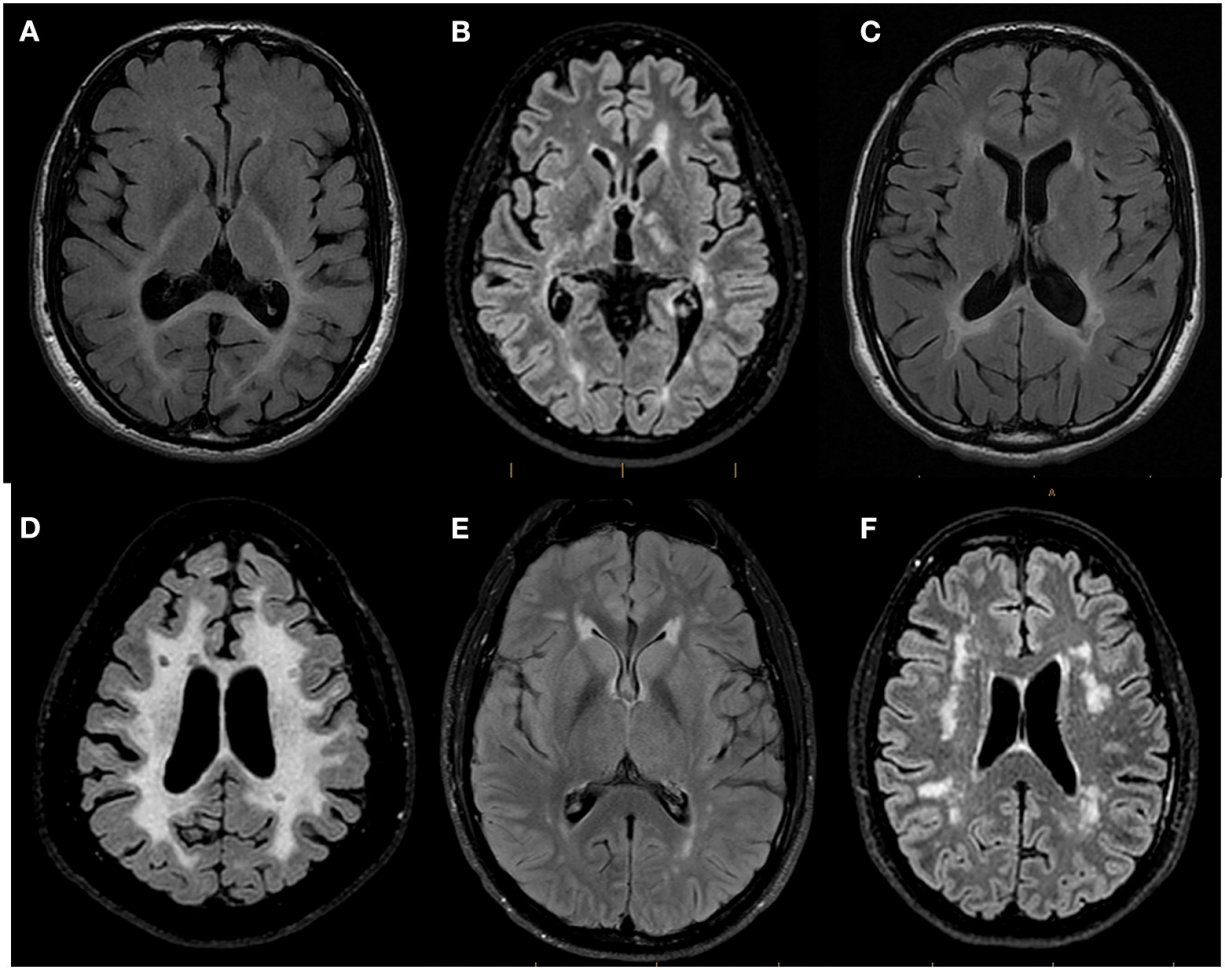
Representative Brain MRI of patients referred to the WM Rounds according to their pattern of involvement. Representative axial FLAIR T2-weighted brain MR images of patients referred to the WMR according to their pattern of involvement. **(A)** 44-year-old man finally diagnosed with Peroxisomal Biogenesis Disorder showing confluent symmetric white matter abnormalities predominant in the posterior regions and affecting the splenium of the corpus callosum and the posterior limb of the internal capsules; **(B)** 29-year-old man diagnosed with X-linked Charcot-Marie-Tooth Disease and MS showing multifocal white matter lesions compatible with MS plaques; **(C)** 43-year-old man with suspected genetic MS mimicker showing bilateral periventricular white matter changes with involvement of the splenium of the corpus callosum and focal T2 hypointense lesions adjacent to the periventricular white matter; **(D)** 44-year-old man (no final diagnosis) with confluent symmetric white matter involvement in which focal T2-hypointense lesions suggestive of MS plaques were identified; **(E)** 38-year-old man with CADASIL (patient vignette no. 1) showing multifocal white matter abnormalities and anterior symmetric periventricular involvement; **(F)** 58-year-old woman (patient vignette no. 2) showing multifocal lobar white matter abnormalities.

The MRI pattern of white matter involvement was interpreted as confluent and mostly symmetric in 25 patients (25/74; 33.8%), thus suggesting a genetic etiology (4, 11), and multifocal in 49 patients (49/74; 66.2%) (Figure 3). Of these 49 subjects, the MRI abnormalities suggested an underlying inflammatory process in 21 cases (21/49; 42.8%), with lesions compatible with MS plaques in 10 subjects (10/49; 20.4%). In 28 subjects with multifocal white matter involvement (28/49; 57.2%), the MRI features suggested either small vessel diseases (hereditary, given the absence of cerebrovascular risk factors in the presented subjects) or other non-inflammatory non-vascular diseases.

### Definite diagnosis

**Figure 4:**
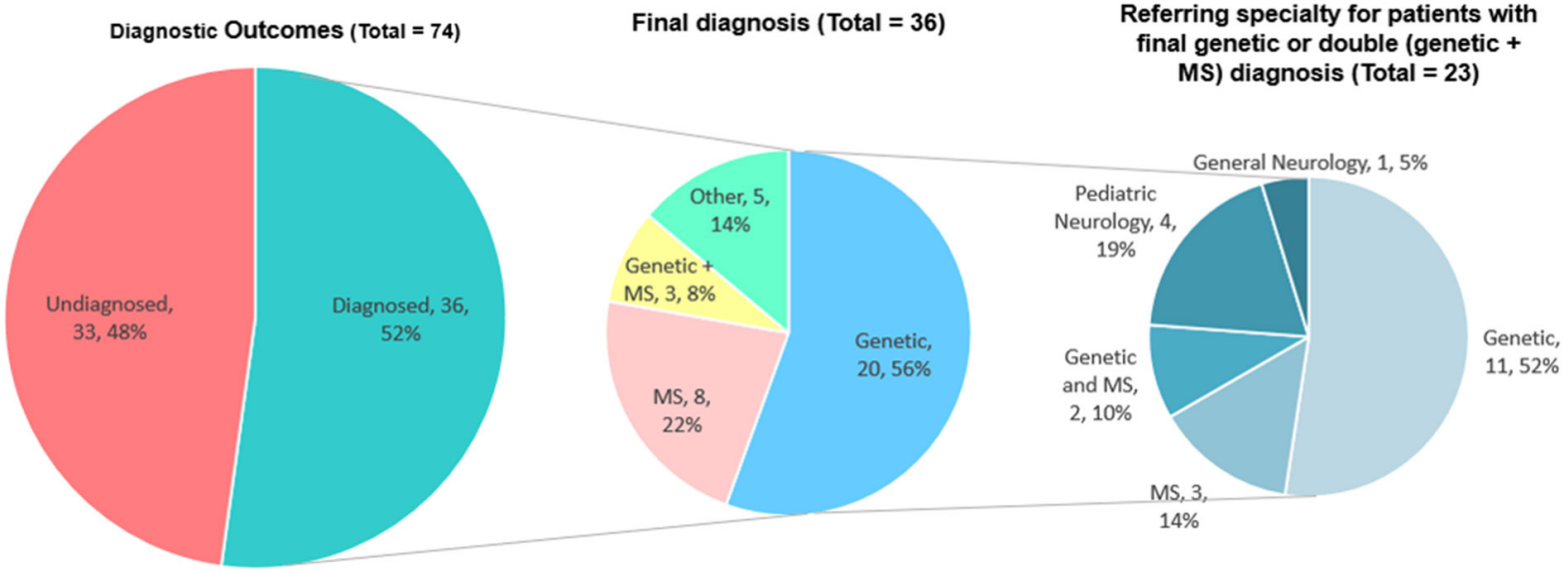
Definite diagnoses in the cohort of 74 subjects presented at the WM Rounds. The data of patients with definite genetic diagnoses are presented according to the subspecialty of their referral physician (right).

We were able to retrospectively collect information on the diagnosis of 69 patients (93.2% of the total 74), while the remaining 5 subjects were lost to follow up (6.8%). Thirty-six patients (36/69; 52.2%) eventually received a definite diagnosis, while 33 (47.8%) remain undiagnosed. Of the 36 diagnosed patients, 20 had a genetic disease, eight had MS, three had a double diagnosis of MS and a genetic condition, and five had other diagnoses (Figure 4).

Table 2 reports the details about the final diagnosis and the association between the radiological interpretation performed during the WM Rounds and the definite diagnosis. Table 3 includes the clinical and mutational data of patients who received a definite genetic diagnosis.

**Table 2:** The definite diagnosis of the 74 patients presented at the WM Rounds according to the interpretation of the MRI findings.

	**Genetic**	**MS**	**Inflammatory not MS**	**Double diagnosis MS/genetic**	**Other**	**No final diagnosis**	**Not available**
Multifocal inflammatory (*n* = 21)	–	7 (33.3%)	2 (9.5%)	2 (9.5%)	–	9 (42.8%)	1 (4.8%)
			Susac syndrome	CMT 1A (1)			
				X-linked CMT (1)			
Multifocal not inflammatory (*n* = 28)	9 (32.1%)	1 (3.6%)	–	1 (3.6.%)	2 (7.2%)	12 (42.8%)	3 (10.7%)
	Mitochondrial disorder (4)			Mitochondrial disorder	Astrocytoma (1)		
	*COL4A1*-related disorder (1)				Multiple cavernoma (1)		
	CADASIL (2)						
	Hypomelanosis of Ito (1)						
	*FOXC1*-related disorder (1)						
Confluent genetic (*n* = 25)	11 (44%)	–	–	–	1 (4%)	12 (48%)	1 (4%)
	Alexander disease (1)				Alcohol-related leukoencephalopathy		
	*COL4A2*-related disorder (1)						
	*FOXC1*-related disorder (1)						
	PBD (1)						
	SCA15 (1)						
	*POL3RA*-related leukodystrophy (1)						
	X-linked ALD (1)						
	SPG7 (1) *SAMD9L*-related disorder (3)						

CMT, Charcot-Marie-Tooth disease; CADASIL, Cerebral autosomal dominant arteriopathy with subcortical infarcts and leukoencephalopathy; PBD, Peroxisomal Biogenesis Disorder; SCA, spinocerebellar ataxia; ALD, adrenoleukodystrophy.

**Table 3:** Clinical and mutational characteristics of the 23 patients with a definite genetic diagnosis.

**Family/Subject**	**Diagnosis**	**Gene**	**Mutation**	**Age of Onset**	**Age of diagnosis**	**Main clinical features**
A.1	MS and Charcot-Marie-Tooth Disease	*GJB1*	p.Arg15Trp	18	20	N, CRB, Vis, V
B.1	Hereditary Cerebral Small Vessel Disease	*COL4A1*	p.Gly1245Val	36	38	M, V
C.3	Peroxisome Biogenesis Disorder	*PEX16*	c.148+5G>A (splice site affected)	8	49	At, V
D.1	Mitochondrial encephalomyopathy	*MT-CYB*	m.15152G>A; p.Gly136Ser	adult-onset	55	LA, M, My, Ex Int
D.2	MS and mitochondrial encephalomyopathy	*MT-CYB*	m.15152G>A; p.Gly136Ser	36	41	TIA, M, My, Ex Int
D.3	Mitochondrial encephalomyopathy	*MT-CYB*	m.15152G>A; p.Gly136Ser	31	32	LA, M, My, Ex Int
D.4	Mitochondrial encephalomyopathy	*MT-CYB*	m.15152G>A; p.Gly136Ser	adult-onset	55	M, My, Ex Int
E.1	MS and Charcot-Marie-Tooth Disease	*CMT1A*	*CMT1A* dup	38	51	N, V, Vis
F.1	Adult-Onset Ataxia With Neuropathy and White Matter Abnormalities	*SAMD9L*	p.His880Arg	63	68	At
F.2	Adult-Onset Ataxia With Neuropathy and White Matter Abnormalities	*SAMD9L*	p.His880Arg	8	35	At
F.3	Adult-Onset Ataxia With Neuropathy and White Matter Abnormalities	*SAMD9L*	p.His880Arg	8	35	At
G.1	CADASIL	*NOTCH3*	p.Cys108Phe	38	45	M, Vis
H.1	POLR3A-related leukodystrophy	*POL3A*	p.Arg694Cys, p.Thr1007IIe	15	27	Am, LD, Myopia
I.1	Hereditary small vessel disease	*COL4A2*	p.Gly800Arg	childhood	59	LD, Mi, Numb
J.1	X-linked Adrenoleukodystrophy	*ABCD1*	n/a	20	51	At, Numb, Vis
K.1	Hereditary small vessel disease	*FOXC1*	p.Arg127His	congenital	24	Aud, M, Vis
L.1	CADASIL	*NOTCH3*	p.Cys49Tyr	21	38	Mi, Numb, Vis
M.1	Mitochondrial encephalopathy	*TUFM*	n/a	37	42	LA, Aud
N.1	Hereditary Spastic Paraplegia type 7	*SPG7*	p.Gly349Ser, p.Gly666Arg	35	50	At, SP, Vis
O.1	Spinocerebellar Ataxia 15	*ITPR1*	p.Val240Met	19	25	At, My
P.1	Alexander disease	*GFAP*	n/a	adult-onset	30	At
Q.1	Hypomelanosis of Ito			congenital	25	Mi
R.1	Leigh Syndrome	*ATP6*	m.9176T>C, p.Leu217Pro	21	21	Severe encephalopathy

Aud, auditory deficit; Am, primary amenorrhea; At, ataxia; CADASIL, Cerebral autosomal dominant arteriopathy with subcortical infarcts and leukoencephalopathy; CRB, cerebellar symptoms; Ex Int, exercise intolerance; LA, lactic acidosis; LD, learning disabilities; M, migraine; My, myoclonus; N, neuropathy; Numb, numbness; SP, spastic paraparesis; TIA, transient ischemic attacks; V, vertigo and/or dizziness; Vis, visual symptoms.

### Definite diagnosis according to MS referrals and the WM Rounds' opinion

Given that several adult genetic leukoencephalopathies may mimic MS, we looked at the definite diagnosis as compared to the WM Rounds' opinion for patients initially assessed by MS specialists. Of the 34 patients referred from an MS specialist, either alone or in collaboration with other specialists, the WM Rounds considered a probable genetic disease in 20 (20/34; 58.8%). From these 20 subjects, a genetic diagnosis was confirmed in five cases (5/34; 14.7%), MS was finally diagnosed in 2 (2/34; 5.9%) and the remaining 13 (13/34; 38.2%) are still without a definite diagnosis and undergoing further genetic investigations. MS was considered the most likely diagnosis in eight subjects referred by MS specialists (8/34; 23.5%) [confirmed in 5 (14.7%), without definite diagnosis in 3], other inflammatory white matter diseases in 3 (3/34; 8.8%) (one confirmed Susac Syndrome, one diagnosed with MS, one without definite diagnosis), and a non-inflammatory, non-genetic diagnosis in two subjects (confirmed in both) (2/34; 5.9%). A double diagnosis of MS and a genetic condition was suspected and later confirmed in one subject (1/34; 2.9%).

To further illustrate the WM Rounds' experience, we include two clinical vignettes of patients discussed during past meetings.

#### Patient 1

A 38-year-old man with a 17-year history of episodes of hemi-body numbness followed by migraines contacted us after reading an article about the WM Rounds. His clinical presentation was a transitory episode of expressive aphasia and visual blurring at the age of 21. Other two episodes of hemi-body numbness, visual blurring, and speech deterioration, followed by headaches, occurred in the following 5 years. The patient was clinically suspected to have Cerebral Autosomal Dominant Arteriopathy with Subcortical Infarcts and Leukoencephalopathy (CADASIL), but *NOTCH3* single gene sequencing (exons 3–6) resulted negative. Subsequently, the episodes increased in frequency to 2–3 times per year and worsened further in the 3 months prior to the most recent consultation when he had a total of eight episodes. Stress, lack of sleep, the winter season, and extreme heat triggered the episodes. He described feeling “inflamed, stiff, and less coordinated” in between episodes. Brain MRI documented multifocal supratentorial periventricular and subcortical white matter abnormalities and raised the suspicion of MS, which was not supported by CSF studies.

His review of systems revealed that he had tinnitus for the past few months, mild hearing loss at high frequency, fatigue, photosensitivity with no decrease in vision, and dizziness. His neurological examination was unremarkable except for decreased vibration and temperature in the right hemi-body. His family history was negative for neurological diseases.

The participants of the WM Rounds agreed that the patient's symptoms were reminiscent of CADASIL's classic episodes of migraine with aura. The review of the brain MRI highlighted the presence of multifocal T2 hyperintensities in the temporal lobes and symmetric anterior periventricular lesions (Figure 3E). These findings were considered typical for CADASIL, although the lack of involvement of the basal ganglia, thalamus, and of the brainstem were not. Despite the previously negative result, the WM Rounds recommended repeating the *NOTCH3* gene sequencing by including all exons. A heterozygous, *NOTCH3* disease-causing mutation (c.146G>A; p.Cys49Tyr) was documented. The variant is a known pathogenic disease mutation (ClinVar ID 447786) located in exon 2. The patient was therefore diagnosed with CADASIL.

#### Patient 2

A 58-year-old woman presented with an almost two-decade-long history of slowly progressive chronic headaches, lower extremity numbness and stiffness, and burning sensations. Fibromyalgia was initially suspected, and amitriptyline was started, without any clinical improvement. As her condition deteriorated, she also developed urinary retention, sleep apnea, and increased fatigue. Brain MRI revealed multifocal white matter abnormalities in the corona radiata and periventricular regions (Figure 3F). She was then transferred to the MS clinic, under suspicion for primary progressive MS (PPMS), which was however never supported by CSF analysis. The patient's past medical history included chronic constipation and infertility; she had no risk factors for cerebrovascular diseases. The patient's sister presented similar symptoms, and, in addition, the family history was positive for systemic lupus erythematosus.

On examination she had brisk deep tendon reflexes (3+) and mild dysmetria. Vibration sense was slightly reduced at the toes, and tandem gait was difficult.

During the WM Rounds, the MR images of the patient and her sister were reviewed. All participants agreed that MS was unlikely and that the imaging findings were more suggestive of a genetic small vessel disease. They recommended to perform a Next Generation Sequencing panel for the genes known to be associated with genetic leukoencephalopathies (220 genes, as well as mitochondrial DNA). However, the results of the panel were negative. This patient was subsequently re-discussed at the WM Rounds, and it was recommended that the patient and her sister be enrolled in a research study on undiagnosed genetic white matter disorders to undergo whole exome sequencing, the results of which are currently pending.

## Discussion

**Figure 5:**
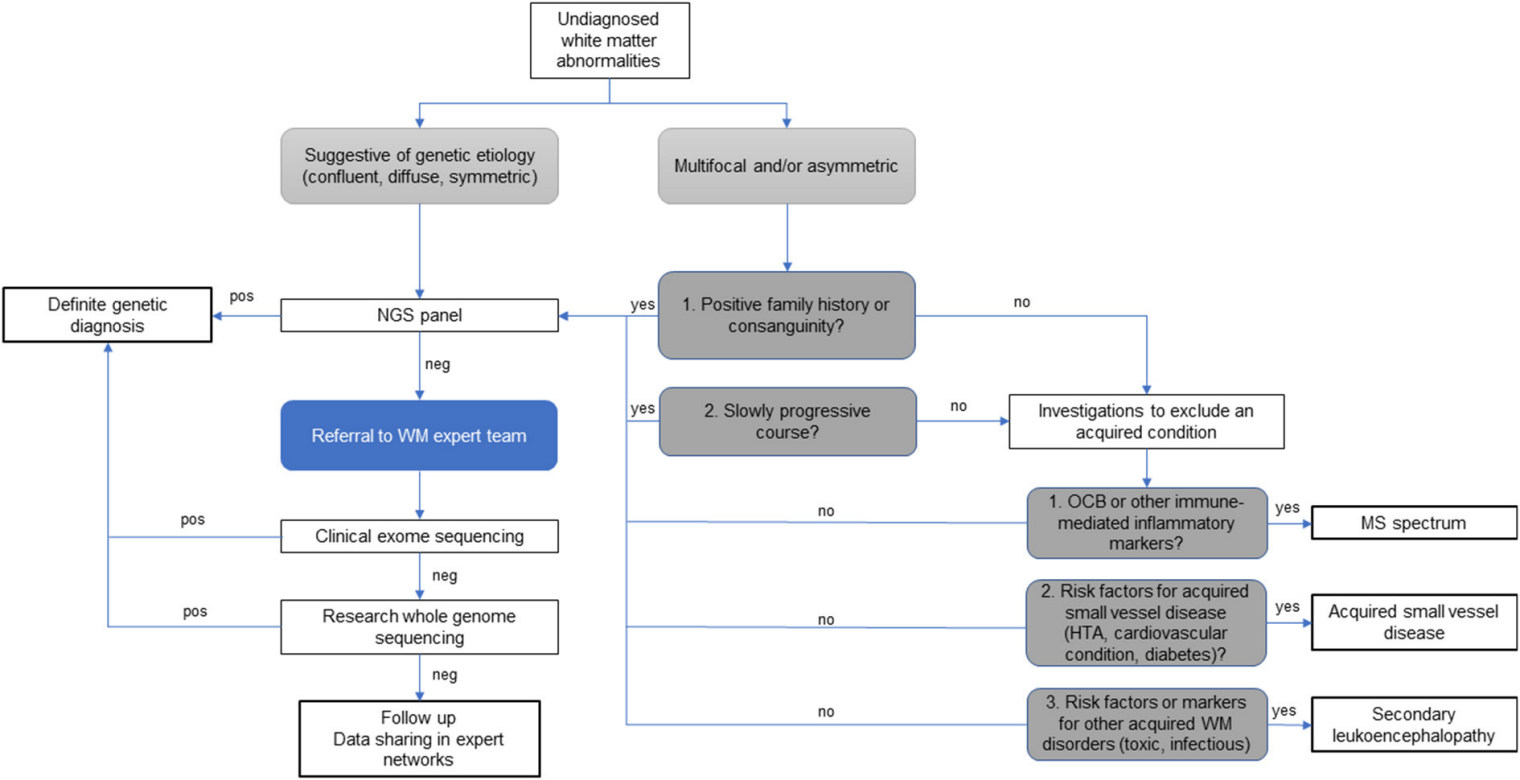
Diagnostic algorithm for patients with atypical undiagnosed white matter abnormalities. *Legend*: HT, hypertension; NGS, next generation sequencing; OCB, oligoclonal bands; WM, white matter; pos, indicates the identification of verified disease-causing mutations; neg, indicates when the data analysis is negative or inconclusive.

The study of rare neurological disorders relies heavily on collaborative networks of specialists that share their experience on limited data from the relatively few affected patients often scattered around the world. In this sense, rare white matter disorders and their overlap with atypical MS pose an added challenge, given that both radiological, clinical, biochemical, and genetic data need to be considered to establish a definite diagnosis. Therefore, in this sub-field, multidisciplinary discussions among experts in white matter disorders are extremely valuable and should be integrated in the diagnostic process and management of atypical and rare white matter diseases presentations, as illustrated in Figure 5, and supported by the literature (10, 15, 16).

Our WM Rounds have evolved into a stronger, continuously expanding collaborative network of scientists and physicians who work on adult genetic white matter diseases and atypical acquired demyelinating disorders. Interestingly, the still ongoing COVID-19 pandemic and the consequent conversion of mixed in-person and web meetings to full online formats had a considerably positive impact on the broadening of this network. The advantages of these regular meetings are evident at multiple levels. Patients who remain without a definite diagnosis can benefit from the experience and insight of specialists from other centers that they otherwise would not have access to. Participants can recognize clinical similarities between a presented patient and their own, with important implications in their current and future clinical practice. Finally, the identification of multiple patients with shared rare phenotypes across different centers enables collaborative studies and inspire future research.

**Figure 6:**
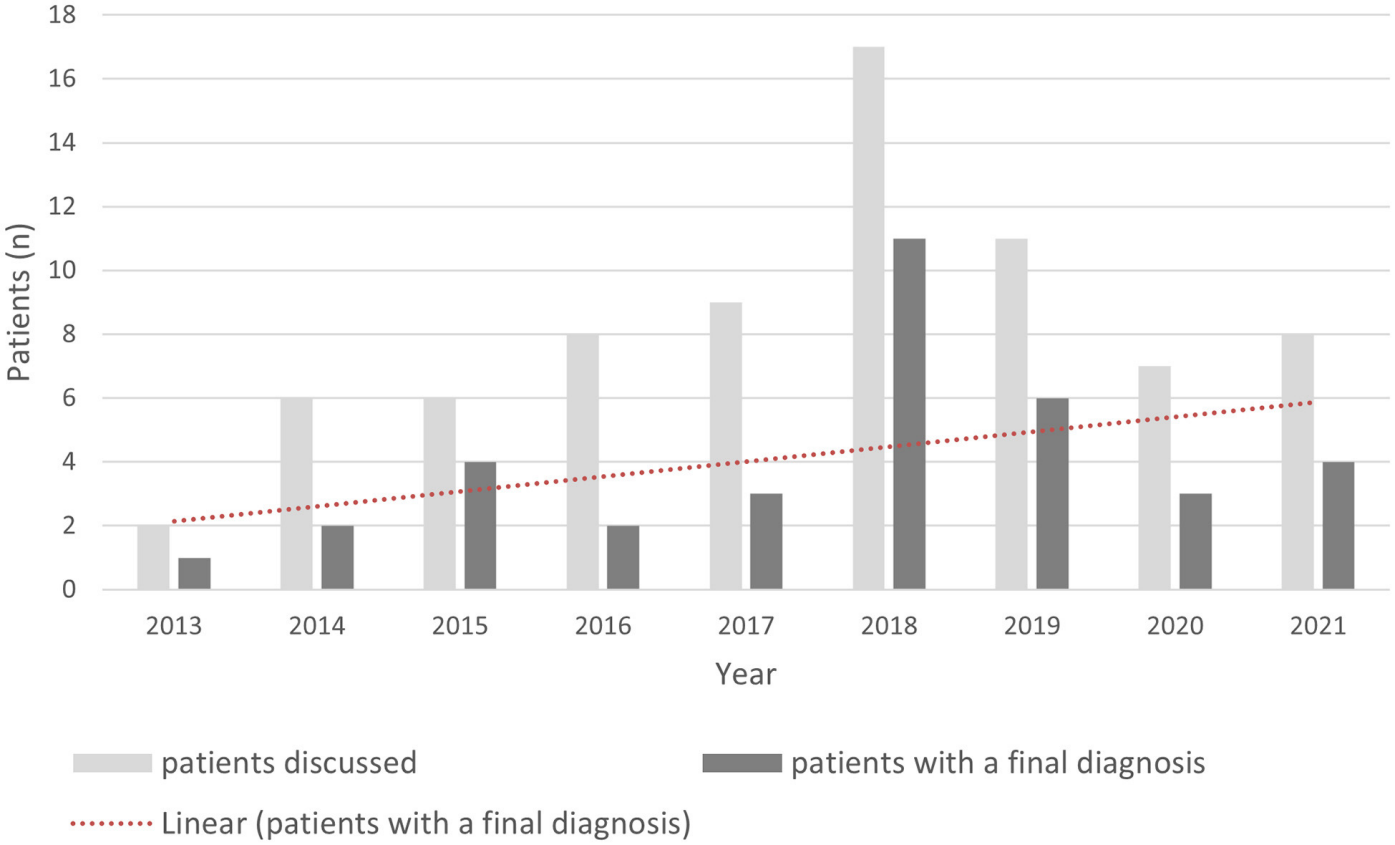
Rate of diagnosis according to the year of presentation at the WM Rounds.

During the first nine years of activity of the WM Rounds, we discussed more than 70 subjects. These discussions have ultimately led to diagnosis in more than half of the cases (52.2%). Figure 6 illustrates the rate of diagnosis per year and its trend across the years, which reflects the improvement of the network's diagnostic skills as well as the availability of newer and more sophisticated diagnostic techniques. This rate of diagnosis is high when compared to published cohorts of adult patients with undiagnosed genetic white matter disorders of probable genetic origin (2–4).

This network has helped physicians and scientists tackle diagnostic challenges specific to the field of adult white matter diseases. In contrast to pediatric forms, adult genetic leukoencephalopathies have a much higher risk of misdiagnosis with acquired demyelinating disorders, specifically MS (17). This is the case for forms with multifocal white matter involvement, which represented 66.2% of our cohort. In general, MS is known to be associated with high risk of misdiagnosis, as the condition has no straightforward disease-specific test, and it is usually a diagnosis of exclusion (18). The distinction between MS, especially the primary progressive forms, and adult genetic leukoencephalopathies often largely depends on the interpretation of their MRI patterns (19, 20). This becomes particularly challenging when presented with patients with suspected MS and atypical MRI findings or subjects with possible genetic disease and multifocal white matter involvement. In our cohort, the identification of lesions with features compatible with MS plaques was indicative of the definite diagnosis of MS even when cases presented atypically. However, the rate of patients with multifocal abnormalities of suspected inflammatory origin who remained without a definite diagnosis is approximately 50%, similar to what observed in the sub-groups of patients with confluent and multifocal non-inflammatory patterns of white matter involvement (2–4). These findings suggest that MRI interpretation is crucial to orient the diagnostic process of white matter diseases, but that adult white matter disorders of unknown origin represent an important issue that should be addressed with targeted research studies.

Approximately half of patients discussed at our WM Rounds were referred by MS specialists. This finding further confirms that genetic MS mimickers represent a diagnostic challenge in undiagnosed white matter disorders. In half of the subjects referred by MS specialists (17/34), we were able to establish a definite diagnosis which included genetic disorders, confirmed MS, other inflammatory disorders, and non-inflammatory non-genetic conditions. We know that MS misdiagnosis accounts for approximately 10% of patients seen in MS clinics (18). We believe that collaborative work between MS specialists, neurologists with expertise in genetics and imaging experts increases diagnostic yields and avoids erroneous diagnosis, as demonstrated by our rounds.

Infections, tumors such as CNS lymphomas and gliomatosis cerebri, iatrogenic leukoencephalopathies, inflammatory disorders, and acquired small vessel diseases are also in the differential diagnosis of adult genetic leukoencephalopathies (3), often due to overlapping non-specific clinical presentation. In our cohort, these diagnoses represented a minimal fraction of the discussed patients.

Approximately 48% of the patients discussed at the WM Rounds remain today without a definite diagnosis and for many of them we suspect an underlying genetic disorder. The challenge of promptly diagnosing a genetic leukoencephalopathy in adult subjects is further compounded by the difficulties in obtaining sufficient information about family history and available DNA from first-degree relatives for testing due to the patients' age. Some of the subjects who are still without a diagnosis benefit from the referral to research studies and are now undergoing genetic analyses in the research context. Networks like the WM Rounds facilitate the identification of potential participants for research studies and provide a basis for future multicenter studies aimed to identify novel genetic leukoencephalopathies and MS mimickers.

## Conclusion

The WM Rounds is a multidisciplinary international network of physicians and scientists that gather remotely every month to discuss challenging or atypical patients with suspected adult-onset genetic leukoencephalopathies. The case discussions have contributed to 52.2% patients ultimately receiving a new diagnosis or having an atypical diagnosis confirmed, in a field of rare diseases where misdiagnoses are common. The experience of the WM Rounds demonstrates the benefit, for patients, scientists, and physicians of a given rare disease field, in regularly having case discussions together to accelerate the diagnostic process, to learn and spread knowledge, and to advance research. Ultimately, it could serve as a model for other rare diseases and complex patients' management.

## Collaborators (White Matter Rounds Network)

Moogeh Baharnoori, Michaela Barbarosie, Philippe Beauchemin, Maria Daniela D'Agostino, Etienne de Villers-Sidani, Pierre Duquette, Zehra Isik Hasiloglu, Manu Jokela, Stephanie R. Keller, Yves Lapierre, Myriam Levesque-Roy, Mika Martikainen, Johanna Ortiz Jimenez, Yann Nadjar, Massimo Pandolfo, John Peters, David Pitt, Mathilde Renaud, Guy Rouleau, Jennifer P. Rubin, Lisa Sabella, Eric Shoubridge, Yannis Trakadis, Ana Cristina Wing, Anna Szekely.

## Data availability statement

The datasets presented in this article are not readily available due to sensitivity reasons because they may contain potentially identifying information. Requests to access the datasets should be directed to the corresponding author, RL.

## Ethics statement

Ethical review and approval was not required for the study on human participants in accordance with the local legislation and institutional requirements. Written informed consent for participation was not required for this study in accordance with the national legislation and the institutional requirements. Written informed consent was obtained from the individual(s) for the publication of any potentially identifiable images or data included in this article.

## Author contributions

YH and RL contributed to conception and design of the study. A-MT, RL, and YH organized the database. YH retrospectively analyzed the data and drafted the manuscript. PG, RM, SV, GF, MS-D, HB, LP-L, LA, AS, MZ, CR, EL, GL, GM, GB, JA, and BB contributed to the collection and interpretation of the data. EM, CM-R, NW, JD, and DT contributed to the interpretation of radiological data. All authors contributed to manuscript revision, read, and approved the submitted version. All authors contributed to the article and approved the submitted version.

## Funding

EL has served on advisory boards for Genentech, TG Therapeutics, and Janssen in the last 24 months. She has received research support from Genentech, NIH K23107624, KL2 TR001862, Race to Erase MS and the Robert Leet and Clara Guthrie Patterson Trust. LA has obtained institutional research funding from Genzyme and Merck, and compensation for lectures and advising from Novartis, Sanofi Genzyme, Merck, Biogen, Roche and Janssen. PG has received honoraria for consulting, speaking and advisory board participation from Actelion, Alexion, Biogen Idec, Bristol Myers Squibb-Celgene, EMD Serono, Genzyme-Sanofi, Innodem Neurosciences, Novartis, Pendopharm, F. Hoffmann-La Roche, and Teva Neuroscience. He has also received research support from EMD Serono, Pendopharm, and F. Hoffmann-La Roche. SV has served on advisory boards for Biogen Idec and has received speaker compensatoin from EMD Serono. GB has received a Clinical Research Scholar Junior 1 Award from the Fonds de Recherche du Québec—Santé (FRQS) (2012–2016), the New Investigator Salary Award from the Canadian Institutes of Health Research (CIHR) (2017–2022) and the Clinical Research Scholar Senior Award from the FRQS. JA received research support from Hoffmann- La Roche Limited. RP has received a Research Scholar Junior 1 Award from the Fonds de Recherche du Québec- Santé (FRQS) and research support from the Canadian Radiological Foundation, Hoffmann-La Roche Limited and funds for open access publication fees from the Tenenbaum Open Science Institute of the Montreal Neurological Institute.

## Conflict of interest

The authors declare that the research was conducted in the absence of any commercial or financial relationships that could be construed as a potential conflict of interest.

## Publisher's note

All claims expressed in this article are solely those of the authors and do not necessarily represent those of their affiliated organizations, or those of the publisher, the editors and the reviewers. Any product that may be evaluated in this article, or claim that may be made by its manufacturer, is not guaranteed or endorsed by the publisher.
